# Performances and Biosensing Mechanisms of Interdigitated Capacitive Sensors Based on the Hetero-mixture of SnO_2_ and In_2_O_3_

**DOI:** 10.3390/s20216323

**Published:** 2020-11-06

**Authors:** Akhil Chandran Mukkattu Kuniyil, Janez Zavašnik, Željka Cvejić, Sohail Sarang, Mitar Simić, Vladimir V. Srdić, Goran M. Stojanović

**Affiliations:** 1 Faculty of Technology, University of Novi Sad, Bulevar Cara Lazara 1, 21000 Novi Sad, Serbia; akhisreekuttan@gmail.com (A.C.M.K.); srdicvv@uns.ac.rs (V.V.S.); 2 Jozef Stefan Institute, Jamova 39, SI 1000 Ljubljana, Slovenia; janez.zavasnik@ijs.si; 3 Faculty of Sciences, University of Novi Sad, Trg Dositeja Obradovića 3, 21000 Novi Sad, Serbia; zeljka.cvejic@df.uns.ac.rs; 4 Faculty of Technical Sciences, University of Novi Sad, Trg Dositeja Obradovića 6, 21000 Novi Sad, Serbia; sohail@uns.ac.rs (S.S.); sgoran@uns.ac.rs (G.M.S.); 5 Faculty of Electrical Engineering, University of Banja Luka, Patre 5, 78000 Banja Luka, Bosnia and Herzegovina

**Keywords:** SnO_2_-In_2_O_3_ composites, self-resonant frequency, electrochemical impedance analysis, biosensing

## Abstract

This study aims to discuss the synthesis and fabrication of SnO_2_-In_2_O_3_-based thick-films and their biosensing applications. The structural characterization of SnO_2_-In_2_O_3_ nanocomposites was performed using X-ray diffraction, Raman spectroscopy and transmission electron microscopy. Furthermore, the screen-printing technology was used in the fabrication of conductive electrodes to form an interdigitated capacitive structure, and the sensor layer based on the mixture of SnO_2_ and In_2_O_3_. Moreover, the sensing performance of the developed structure was tested using *Pseudomonas aeruginosa* (*P. aeruginosa*) and *Staphylococcus aureus* (*S. aureus*) bacteria. In addition, the validation of sensing characteristics was performed by electrochemical impedance spectroscopic and self-resonant frequency analysis. Finally, the sensing properties were analyzed for two consecutive days, and changes in both *P. aeruginosa* and *S. aureus* pathogens growing media were also studied.

## 1. Introduction

With launching the concept of the Internet of Everything and with the possibility to process big data sets, sensors, and particularly biosensors, are gaining increasing attention. This is because these sensors can improve the quality of our life [1]. The development of nanomaterials has enabled the manufacturing of biosensors with better characteristics. Among other materials, metal oxides (MOs) have been widely used in the fabrication of cost-effective sensors for numerous applications [2]. Thin- and thick-film technologies can be used for mass-producing MO-based gas sensors, which are robust, durable and can be applied in portable devices. Tin oxide (SnO_2_) and indium oxide (In_2_O_3_) are wide-bandgap semiconductors that typically crystallize in the rutile and cubic structures, respectively [3]. As sensing materials, MOs demonstrate good stability and fast response. Moreover, they are suitable for miniaturization, being compatible with standard fabrication techniques. Alloys consisting of two (or more) metal oxides can lead to significant improvements in sensitivity [4]. Because of that, numerous applications of MO-based heterogeneous sensors have been reported. SnO_2_-In_2_O_3_ structures attain special interest because of the cost-effectiveness of SnO_2_ and also the good conducting nature of In_2_O_3_ material. The SnO_2_-In_2_O_3_ hetero structure causes an increase in oxygen vacancies of the crystal lattice, leading to more structural defectiveness. Hence, this enhances the sensing performance in comparison to a single oxide of SnO_2_ or In_2_O_3_, making it very promising for various sensing applications. For example, a sensor made of tin oxide containing 40 mol% of indium oxide demonstrated a good sensitivity for the detection of CO in the air [5]. It was shown that the ratio of 20 mol% of In_2_O_3_ and 80 mol% SnO_2_ had high sensitivity for the detection of hydrogen in the air [6]. Moreover, a film containing 19% of In_2_O_3_ and the rest SnO_2_ also exhibited high sensitivity to hydrogen [7]. Additionally, the same authors reported the detection of CO in the temperature range from 330 °C to 520 °C using nanostructured films of SnO_2_, In_2_O_3_, and their combinations [8]. Thin films of In_2_O_3_-SnO_2_ nanostructured materials were also analyzed, resulting in films where only one oxide crystallized, whereas the other produced a doping effect that was used to detect different NO_2_ concentrations [9]. Thus, very similar structures of SnO_2_-In_2_O_3_ nanocomposites have shown sensitivities to different mediums. Because of that, there is a need for an in-depth explanation of sensing mechanisms in each application. Moreover, SnO_2_-In_2_O_3_ nanocomposites were developed as sensing materials for the detection of toxic gases such as CO and NOx as well [10]. Analysis showed that SnO_2_-In_2_O_3_ nanocomposites functionalized with Pd can be used for the detection of flammable gases such as butane, but in an operating temperature ranging from 280 °C to 480 °C [11]. The structure containing 3% of In_2_O_3_ exhibited a better response than the SnO_2_ structure for the detection of the toxic indoor air pollutant formaldehyde [12]. It was reported that operating temperatures can be reduced with the addition of a moderate quantity of SnO_2_ to the In_2_O_3_ [13]. There were trials to also include other elements—for example, the addition of small amounts of CeO_2_ to In_2_O_3_ nanocrystals—in order to improve the sensor response, whereas the addition of CeO_2_ to SnO_2_ matrix caused a decrease in the sensor response [14]. Additionally, Pd-SnO_2_ was used as a sensing material for the detection of carbon monoxide, In_2_O_3_ for the detection of nitrogen oxide, Ru-WO_3_ for ammonia, and SnO_2_-ZnO for formaldehyde gases [15]. Several experimental studies demonstrated that parameters such as film thickness, porosity, agglomeration, grain size, area of inter-grain contacts had an influence on the SnO_2_- and In_2_O_3_-based sensors properties [16,17,18]. Nanostructured film with a thickness of 12 nm of hetero-structure of 95% In_2_O_3_ + 5% SnO_2_/ns-Si demonstrated an ability to detect alcohol vapors [19]. Bacterial infections are one of the leading causes of mortality nowadays, and because of that the development of biosensors for the detection of different pathogens has attracted the attention of scientists worldwide [20]. The sensor platform for the detection of *Escherichia coli* and other water-borne pathogens was reported in [21]. Zinc oxide (ZnO) nanorods and graphene nanoflakes were used on an indium-tin oxide substrate. Self-assembled monolayers on Au electrodes for the detection of *Staphylococcus aureus* (*S. aureus*) at the electrode were reported in [22]. Lam et al. improved the specificity and sensitivity of a biosensor by implementing the interdigitated array microelectrodes [23]. The stability of SnO_2_-In_2_O_3_ hetero-structures was analyzed in [5,24]. It was shown that In_2_O_3_ acts as a barrier against grain growth and loss in surface area. Moreover, the enhancement of the phase stability of the resulting nanocomposites was reported [5,24]. However, even though MO-based sensors are highly required as biosensors, there is still a lack of evidence explaining the sensing mechanisms in the case of various bacteria.

The development of bacterial detection systems is gaining significant attention in scientific and professional communities because bacterial infections represent a serious threat to humans. Due to the existence of different bacteria species, numerous efforts are being made for the development of tools for bacteria discrimination based on analytical instrumentation (tandem gas chromatography-mass spectrometry [25,26], secondary electrospray ionization mass spectrometry [27]), or ion mobility spectrometers [28]. Moreover, the effect of growth medium on the bacterial pathway was analyzed [29]. Additionally, the influence of different antibiotics on the inhibition of bacteria grown in different media was reported [30].

In this paper, we advance the state of the art with biosensors composed of electrodes in the form of interdigitated planar capacitor and sensitive layer on the top, made from three different mixtures of SnO_2_ and In_2_O_3_. The prepared nanocomposites were used for the fabrication of impedimetric sensors for bacteria detection. The sensing performances were analyzed using two different approaches: (1) the electro-chemical impedance spectroscopic method and (2) self-resonant frequency analysis. The changes in the electrical parameters, such as impedance and capacitance, of SnO_2_-In_2_O_3_ thick-film based sensors were studied, when they were exposed to the *Pseudomonas aeruginosa* (*P. aeruginosa*) and *S. aureus* pathogen media.

The novelty and significance of our work can be summarized as follows: (1) sensing performances of different weight ratios of In_2_O_3_ and SnO_2_ were analyzed to provide evaluation of new materials, which can be very useful for future advances in the field, (2) economical and short development fabrication procedures were presented to enable mass-production of sensors for bacteria detection, (3) conducted comprehensive characterization by means of X-ray diffraction, Raman spectroscopy and transmission electron microscopy were discussed in detail, providing real-world values for specific parameters which are important for material evaluation by other researchers, (4) detailed analysis of sensing mechanisms provides new knowledge for better understanding of MO-based sensors’ behavior in bacterial growth media, (5) differentiation between *P. aeruginosa* and *S. aureus* was obtained with a straight-forward sensor design, and (6) an interface with developed sensors does not require any specific and expensive tool, and furthermore it can be done with simple and portable embedded hardware for impedance measurement, enabling in situ applications. Interdigitated capacitive (IDC) sensors based on a hetero-mixture of SnO_2_ and In_2_O_3_ open up new avenues for various biomedical applications in which reliable bacteria monitoring is needed. The economical fabrication procedure and low complexity of required readout electronics demonstrate the potential to generate innovation in bacteria monitoring to avoid the need for costly equipment.

This paper is organized as follows: in Section 2, the main details regarding the preparation of SnO_2_-In_2_O_3_ composite powders and sensor fabrication are provided. The structural characterization and analysis of sensing performances/mechanism of the fabricated sensors are provided in Section 3. Concluding, Section 4 summarizes the main contributions of the paper and provides directions for our future work in the field.

## 2. Experimental

### 2.1. Used Chemical and Materials

Nanocomposite powders of SnO_2_-In_2_O_3_ have been prepared using pre-synthesized powders of SnO_2_ and In_2_O_3_. Sn(II)Cl_2_ (purity 98%) and In(III)Cl_3_ (purity 99.99% metal basis) were obtained from Alfa-Aesar.

Alumina and Ag/Pd paste were used as sensor substrate and conductive material for electrodes, respectively.

### 2.2. Preparation of SnO_2_-In_2_O_3_ Composite Powder

As mentioned above, nanocomposite powders of SnO_2_-In_2_O_3_ were prepared by using pre-synthesized powders of SnO_2_ and In_2_O_3_. Both of these metal oxide powders were synthesized by following the co-precipitation method, using Sn(II)Cl_2_ and anhydrous In(III)Cl_3_ as the precursor material for SnO_2_ and In_2_O_3_, respectively. Different weight ratios of In_2_O_3_ and SnO_2_ were prepared as combinations of 5:95, 10:90, and 15:85 wt. percentages followed by mixing, grinding, and processes using a planetary ball miller. Six cycles of grinding process continued at 400 rpm using three-millimeter silica balls in 1:3 power-ball ratios in isopropanol as the solvent medium. The final product was kept overnight for evaporation at room temperature. The obtained powder was used for further fabrication of the proposed thick-film sensors.

### 2.3. SnO_2_-In_2_O_3_ Sensor Fabrication

**Figure 1:**
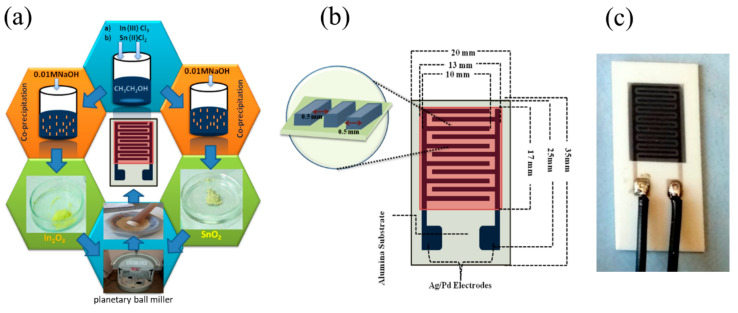
(**a**) Steps involved in synthesis process, (**b**) schematic representation of SnO_2_-In_2_O_3_ nanocomposite-based interdigitated capacitive (IDC) sensor structure, (**c**) fabricated sensor on alumina substrate.

The thick-films of SnO_2_-In_2_O_3_ were fabricated after the following steps of the metal oxide paste preparation. The nanocomposite powder was gently mixed with the binding solution in a mortar; here, a homogeneous mixture of ethyl cellulose and terpineol in 1:9 ratio was used as a binding agent for the paste preparation. The process of mixing of SnO_2_-In_2_O_3_ powder with binding agent solution was continued until obtaining a homogeneous paste. The conductive electrodes in the form of interdigitated (comb) capacitive structure and the sensor layer were fabricated by screen printing technology. Schematic steps of the synthesis process are shown in Figure 1a. The geometrical dimensions of the fabricated interdigitated capacitive (IDC) sensor structure are depicted in Figure 1b. We used synthesized SnO_2_-In_2_O_3_ composite paste as a sensor layer and Ag/Pd paste as conductive materials for electrodes. The structures were sintered at 600 °C and the fabricated sensor can be seen in Figure 1c.

### 2.4. Preparation of Microbial Culture

In this study, we used *P. aeruginosa* and *S. aureus* pathogen cultures as the bulk solution medium to test the sensing performances of the proposed SnO_2_-In_2_O_3_ based sensors. The above-mentioned pathogen colonies were cultured for 24 h on blood agar (HiMedia, Mumbai, India) in sterile tubes. The density of bacterial cells was 0.5 McFarland (MCF) in 4.5 mL of physiological saline using EUCAST standard.

### 2.5. Characterization Techniques and Apparatus

The structural characterization of SnO_2_-In_2_O_3_ nanocomposites was performed using X-ray diffraction (Rigaku MiniFlex 600, Rigaku, Tokyo, Japan), Raman spectroscopy (Thermo Fisher DXR Raman microscope, Waltham, Massachusetts, U.S.) and transmission electron microscopy (TEM, JEM-2100, Jeol Inc., Tokyo, Japan), operating at 200 kV. Electrical impedance and capacitance as a function of frequency were measured using the Impedance Analyzer (HP4194A, Palo Alto, CA, USA).

## 3. Results and Discussion

### 3.1. Structural Characterization

**Figure 2:**
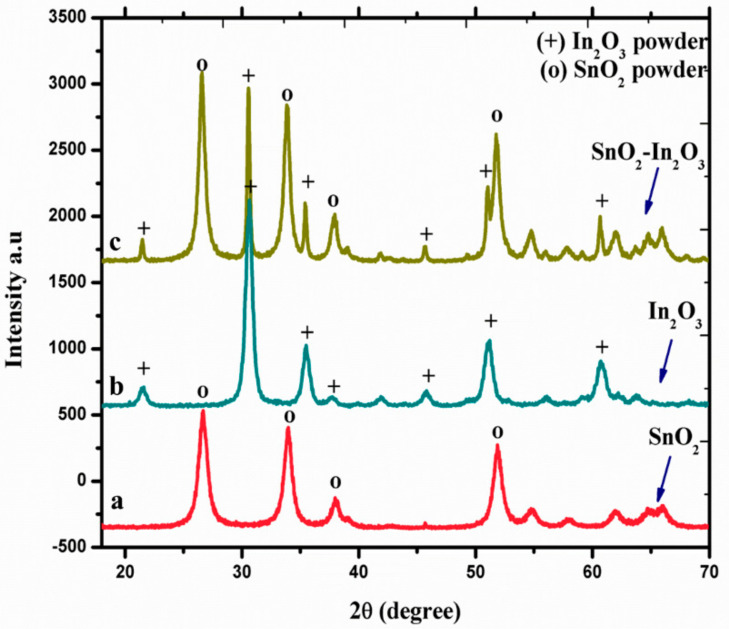
XRD spectra of SnO_2_, In_2_O_3_, and SnO_2_-In_2_O_3_ nano-powders.

X-ray diffraction spectroscopy (XRD) was used to study the crystal phase formation and structural properties of SnO_2_, In_2_O_3_, and SnO_2_-In_2_O_3_ (85:15 wt.%) nanocomposite powder, and these three nano powders were sintered at 600 °C. The diffraction peaks observed in Figure 2 (a line) can be indexed to the tetragonal rutile structure of SnO_2_ (JCPDS No. 77-0450). Figure 2 (b line) shows the diffractions corresponding to body centered cubic phase of In_2_O_3_ with the lattice constant of a = 10.11 Å (JCPDS No. 71-2194). Moreover, no impurities were observed in both a and b lines of Figure 2 diffraction patterns. Figure 2 (c line) represents XRD pattern of SnO_2_-In_2_O_3_ nanocomposite powder. The diffraction pattern confirms that the nanocomposite powder exists with crystal phases of both SnO_2_ and In_2_O_3_.

**Figure 3:**
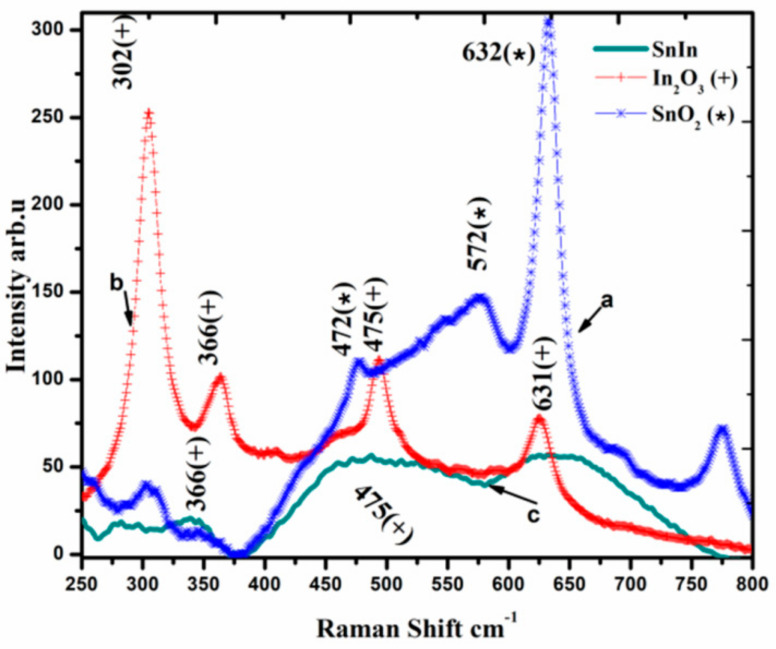
Raman spectra of SnO_2_, In_2_O_3_ and SnO_2_-In_2_O_3_ (85:15 wt%) nano-powders.

Raman spectroscopic analysis of SnO_2_ and In_2_O_3_ powders and SnO_2_-In_2_O_3_ (85:15 wt%) nanocomposite powder is presented in Figure 3. The analysis reveals that SnO_2_-In_2_O_3_ (85:15 wt%) powder occupied binary metal phases. Raman shifts for SnO_2_ powder are at 472, 572 and 632 cm^−1^ (emphasized with “*” sign). Raman shifts for In_2_O_3_ powder are at 366, 475 and 631 cm^−1^ (highlighted with “+” sign). Raman shifts for SnO_2_-In_2_O_3_ nanocomposite powder are observed at 366, 475 (representing In_2_O_3_ metal oxide phase) and at 631 cm^−1^ (showing a presence of SnO_2_ metal phase). Raman shifts at 475 cm^−1^ of In_2_O_3_ metal phase and 631 cm^−1^ of SnO_2_ metal phase shifts were not clearly visible from the result. One can assume that those shifts were merged with the nearest ones.

**Figure 4:**
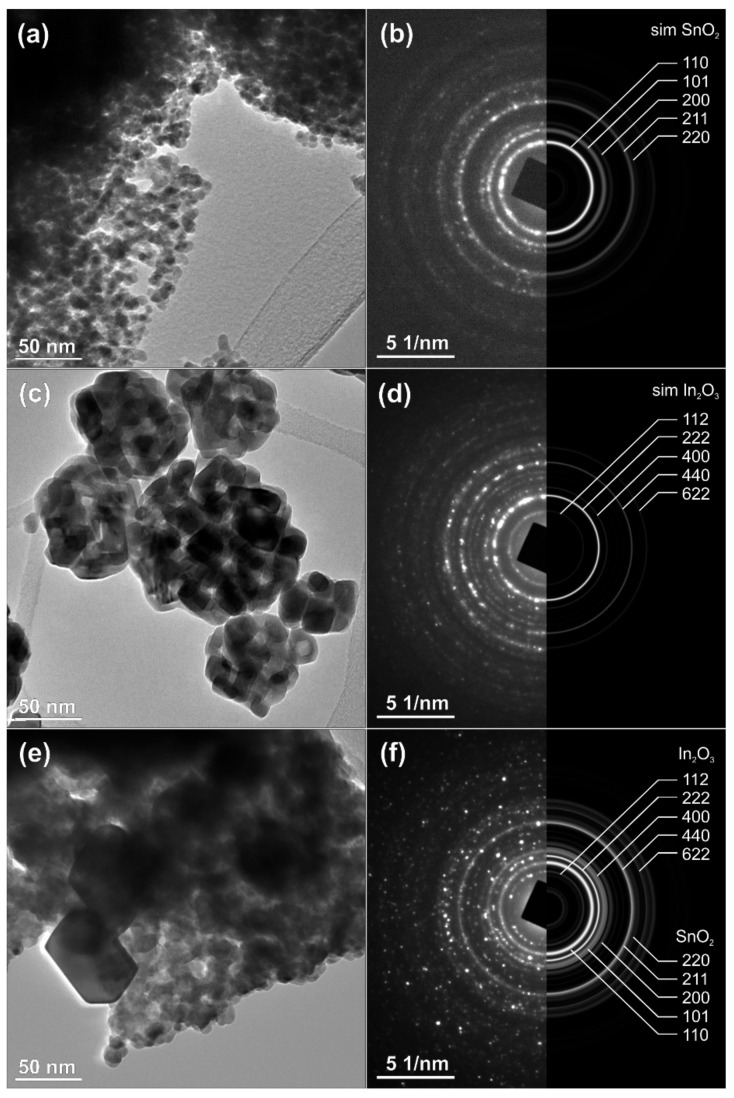
TEM analysis of SnO_2_-In_2_O_3_ nano-composite powder (**a**,**b**) HRTEM and SEAD image of SnO_2_ powder, (**c**,**d**) TEM and SEAD images of In_2_O_3_ powder, (**e**,**f**) TEM and SEAD images of SnO_2_-In_2_O_3_ (85:15 wt%) nano powders, respectively.

The results of TEM analysis of the as-prepared nanopowders of SnO_2_, In_2_O_3_ and the nanocomposite of SnO_2_-In_2_O_3_ (85:15 wt%) are presented in Figure 4. The selected-area electron diffraction (SAED) patterns, collected over multiple particles, correspond perfectly with the simulations calculated from the theoretical data for pure, crystalline tetragonal SnO_2_ (Figure 4a,b) and for cubic In_2_O_3_ (Figure 4c,d), while the nanocomposite corresponds to a mixture of pure phases (Figure 4e,f). The size of the SnO_2_ crystallites is in the range of 10–20 nm, while In_2_O_3_ crystallites are somehow bigger, up to 50 nm; such size distribution is also preserved in the nanocomposite. The pattern confirms the presence of SnO_2_ and In_2_O_3_ crystal phases. These results are in good agreement with the results already presented in Figure 2 and Figure 3.

### 3.2. Analysis of Sensing Performances

#### 3.2.1. Self-Resonant Frequency (SRF) Analysis

**Figure 5:**
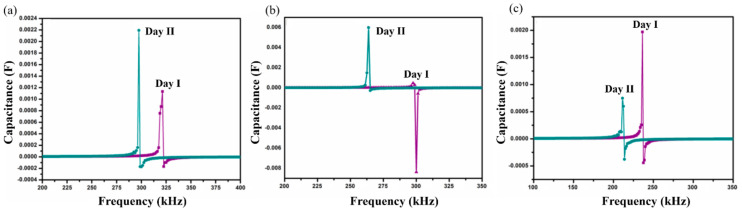
Shift in self-resonant frequency (SRF) in two consecutive days for samples (**a**) SnIn5, (**b**) SnIn10 and (**c**) SnIn15, in *P. aeruginosa* media.

**Figure 6:**
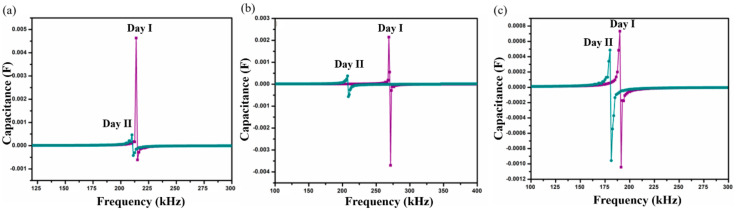
Shift in SRF on two consecutive days for samples (**a**) SnIn5, (**b**) SnIn10 and (**c**) SnIn15 in *S. aureus* media.

Self-resonant frequency (SRF) analysis of SnO_2_-In_2_O_3_ nanocomposite-based sensors was conducted in *P. aeruginosa* and *S. aureus* cultures, used as the bulk sensing medium in order to analyze the sensing performance of the proposed structure. Thick-films of various chemical compositions, namely 95:5, 90:10 and 85:15 weight percentages of SnO_2_ and In_2_O_3_, respectively, were developed to conduct this analysis. The studied samples were labeled as SnIn5, SnIn10, and SnIn15. The capacitance as a function of frequency was measured by means of an Impedance Analyzer (HP4194A), and the SRF when the component makes the transition from capacitive character to inductive was detected. Figure 5 and Figure 6 show SRF responses from SnIn5, SnIn10, and SnIn15 thick-film sensors. The analysis with the same bacterial cultures was conducted for two consecutive days under the same experimental conditions. The main purpose of the developed sensor is to provide fast and reliable bacteria detection. Because of that, experiments were conducted over two days to demonstrate that our sensor is capable of detecting bacteria multiplication without the need for long and complex procedures. It is reasonable to expect that running the experiment for longer than two days will provide the same trend in the change in SRF and capacitance of the interdigitated sensor until the stationary and death phase of the bacterial life cycle.

Figure 5 presents the results obtained when the sensor was exposed to *P. aeruginosa* as a sensing medium. SRF shifts observed on the SnIn5 sample can be seen in Figure 5a. On Day I, SRF was observed at 318 kHz and it was shifted to a lower frequency value of 297 kHz on Day II. Figure 5b,c represent results from SnIn10 and SnIn15, respectively. Similar behavior can be observed, shifting SRF from 297 to 263 kHz in Figure 5b, and from 235 to 211 kHz in Figure 5c, for both Day I and Day II analysis.

**Figure 7:**
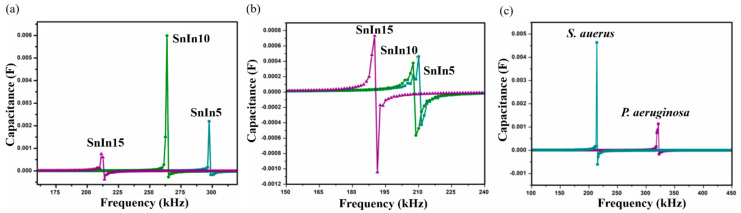
(**a**) SRF observed from *P. aeruginosa*, (**b**) SRF observed from *S. aureus* using SnIn5, SnIn10 and SnIn15 samples, and (**c**) comparison of obtained SRF values from *S. aureus* and *P. aeruginosa*, for sample SnIn5.

The results obtained from *S. aureus* culture are depicted in Figure 6. The results also show the shifting of SRF to lower frequencies on Day II compared to Day I analysis. On Day I, SRF was observed at 213 kHz and it was shifted to a lower frequency value of 210 kHz on Day II, for SnIn5 sample. Figure 6b,c represent the results from SnIn10 and SnIn15, respectively. Similar behavior can be seen in the shifting of SRF from 268 kHz to 207 kHz in Figure 6b, and from 190 kHz to 179 kHz in Figure 6c. The results indicate an increase in the capacitance of the sensing electrode due to the increase in the ionic concentration of sensing media [31]. This increase in the ionic concentration of sensing media is because of the secretion of charged products as their metabolic product. Figure 7 shows a comparative analysis of SRF values obtained from SnIn5, SnIn10, and SnIn15 samples. The values for SRF were 297, 263, and 212 kHz in *P. aeruginosa*, which can be seen in Figure 7a, whereas SRF was identified at 210, 207, and 189 kHz in *S. aureus*, as shown in Figure 7b. It can be concluded that increasing In_2_O_3_ concentration results in the SRF value shifting towards lower frequencies, and the same behavior was observed in both *P. aeruginosa* and *S. aureus* media.

#### 3.2.2. Impedance Spectroscopic Analysis

**Figure 8:**
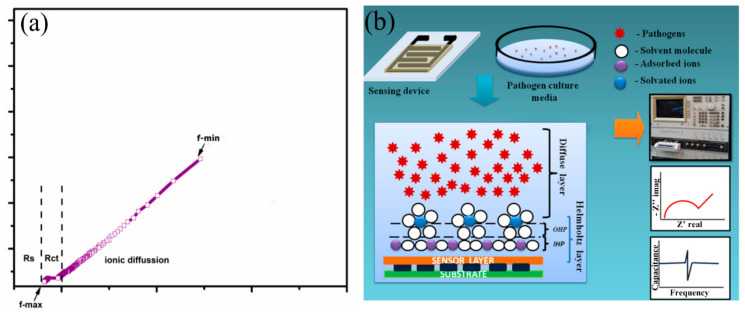
Schematic representation of (**a**) Nyquist plot obtained from SnIn5 thick-film sensor (**b**) site binding model with steps in sensing analysis.

To analyze the sensing characteristics of the developed SnO_2_-In_2_O_3_-based sensors, impedance spectroscopy was used as a non-destructive technique that offers the measurement of impedance response of an applied AC potential. Since the impedance is a frequency-dependent parameter, it can reveal electro-chemical changes that occurred in the analyzed sensor system [32]. The data obtained from an electrochemical system can be exploited using the Nyquist plot and Bode plot analysis [33]. In an electro-chemical reaction, impedance variation in Faradaic reaction can be performed due to various factors, such as: (1) adsorption of reacting molecules; (2) diffusion of ions; and (3) both diffusion and adsorption process. A schematic representation of a Nyquist plot for the structure of SnIn5 (SnO_2_-In_2_O_3_(95:5 wt%)) is illustrated in Figure 8. The plot also explains the solution resistance (Rs), the charge transfer resistance (Rct), and the adsorption/desorption process.

**Figure 9:**
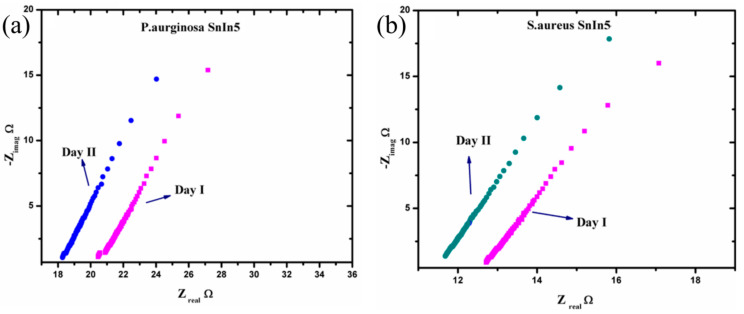
Nyquist plot response from (**a**) *P. aeruginosa* and (**b**) *S. aureus* in two consecutive analysis.

The Nyquist plot consists of one small arc and another uncompleted bigger arc. The diameter of the small arc formed at high frequency is considered as Rct. The uncompleted arc formed at lower frequency representing the adsorption/diffusion process occurred at the electrode-solution interface as illustrated in Figure 8b. According to cite binding theory, when a metal oxide is targeted with an aqueous media, the charged surface group formed on the metal surface. H^+^ and OH^−^ ions from electrolyte adsorbed to the metal surface and form a charge interface between the metal oxide surface and electrolyte. This charged interface, called the double layer capacitance, is also illustrated in Figure 8b. As shown in Figure 8b, the hydroxyl ions and protons were attracted to surface cation and oxygen lattice [34]. The concentration of these potential ions attracted to the metal lattice and surface determine the electrical parameter such as capacitance, resistance, and impedance [35]. In this study, we measured the changes in the ionic concentration in a growing colony of pathogen media. The ionic changes in pathogen media are due to the metabolic secretion during their biological activities. This process leads to changes in the concentration of attracted potential ions, which results in changes to the electrical parameters. Figure 9 represents electrochemical changes that occurred on the SnIn5 sensor in the (a) *P. aeruginosa* and (b) *S. aureus* pathogen environment.

From Figure 9, it can be observed that the diameter of the small arc formed at the high frequency part of the Nyquist plot is slightly reduced on Day II of the analysis compared to Day I. Moreover, the solution resistance has a tendency to decrease on Day II comparing to the data obtained on Day I, in both pathogen media. This can be attributed to the variation in ionic concentration in the pathogen system during the time, and this consequently changes the values of the parameters Rct and Rs. This explains the dependency of charge transfer resistance with the concentration of ions in the bulk media. The Rct and Rs values were extracted from the graphs in Figure 9 and the results are presented in Table 1.

#### 3.2.3. Sensing Mechanisms Analysis

Impedance changes of the sensor immersed in the solution with bacteria occurred because of: (1) metabolites produced by bacterial cells during bacteria growth, and (2) surface changes of the electrodes due to the bacteria attachment. A decrease in impedance with time is a good indicator that the microorganisms are consuming growth media substrates of low conductivity, metabolizing them into ionic products of higher conductivity [36,37]. If impedance decreases with time, it means that capacitance of IDC was increased, which leads to the decreasing of SRF (SRF = 1/(2√(LC))). By adding bacterial cells of *P. aeruginosa* and *S. aureus* into physiological saline, metabolic activities are initiated in which bacteria convert large molecules (polysaccharides, lipids, nucleic acids, and proteins) into smaller units which are more mobile (monosaccharides, fatty acids, nucleotides, and amino acids), and therefore change the ionic composition of the growth media [30]. Therefore, it can be expected that the permittivity (relative dielectric constant value) and conductivity of the solution will change. The change in the ionic composition of the surrounding medium will be reflected in the impedance of the immersed IDC sensor. There are four phases during bacterial growth: lag, log, stationary, and death phase. An increase in the live cell number is mostly in the log phase, where the multiplication of bacteria is extensive. Because of that, there is a decrease in the overall impedance of immersed IDC sensor, as there are an increasing number of bacterial microorganisms that are consuming growth media substrates of low conductivity and metabolizing them into ionic products of higher conductivity [36]. Thus, measuring the capacitance and impedance of immersed sensor in solution with bacterial cells can be used as a sensing mechanism. However, the electrical response of the solution with bacterial cells depends on the shape, size, and type of bacteria that enables differentiation in changes made by the presence of *S. aureus* or *P. aeruginosa*. *S. aureus* is a spherical Gram-positive bacterium, which is immobile and forms grape-like clusters, whereas *P. aeruginosa* is a rod-shaped Gram-negative bacterium and tends to form biofilms that adhere to a variety of surfaces. It has been previously reported that *S. aureus* has almost twice shorter doubling time in comparison to *P. aeruginosa* [38]. In the context of cellular membranes, the Gram-negative bacteria are surrounded by a peptidoglycan cell wall and an outer cell membrane, whereas the Gram-positive bacteria lack an outer cell-membrane [39]. Due to the lack of an outer cell-membrane, the extracellular secretions from Gram-positive bacteria need to pass through only one membrane to reach the extracellular environment [40,41]. Moreover, Gram-positive bacteria have 3–4 times higher values of relative dielectric constant in comparison to Gram-negative bacteria [42]. Based on the above-mentioned statements, it can be concluded that: (1) *S. aureus* is a Gram-positive bacterium, which means that a higher value of the relative dielectric constant is expected in comparison to *P. aeruginosa*. Because of that, in the case of *S. aureus*, a higher relative dielectric constant means that higher capacitance should be obtained, which consequently leads to the lower resonant frequency in comparison to *P. aeruginosa* (this can be seen in Figure 7c); (2) Because of higher concentration (shorter doubling time) of ionic products with higher conductivity, lower impedance should be obtained for *S. aureus* in comparison to *P. aeruginosa*. Lower overall impedance should lead to a lower value of series resistance (Rs) in the case of *S. aureus* in comparison to *P. aeruginosa* (this is proved by the results given in Table 1). (3) The tendency of *P. aeruginosa* to form biofilms and to stick on sensing electrode structure should lead to slower charge transfer kinetics and therefore increased charge transfer resistance (Rct) in comparison to *S. aureus* (this is demonstrated in Table 1).

Importance of developing methods for bacteria discrimination has already been reported. For example, aspiration type ion mobility spectrometer (a-IMS) was used for direct monitoring of the headspace atmosphere above cultures of *Escherichia coli*, *Bacillus subtilis* and *S. aureus* [28,43]. It was shown that each of three bacterial species generate their own chemical profile (released volatile organic compounds), enabling fast and reliable differentiation among pathogens. We think that our approach, based on SRF measurement and impedance spectroscopic analysis, can be combined with an IMS approach for rapid bacteria discrimination. Both approaches are suitable for implementation on portable hand-held embedded hardware. These two reliable approaches can complement each other in specific applications with an increased level of accuracy and reliability.

Evaluation of the sensing performance of the fabricated sensors is usually done by economical aspects of the production process, required sensing area for reliable sensing, as well as sensitivity and selectivity. The developed sensors are based on the IDC structure, which offers benefits such as economical and short development processes followed by reliable and repeatable fabrication procedures. In general, IDC sensors have small dimensions and low power consumption. The proposed sensors have overall dimensions 20 mm × 35 mm, with an active area of sensing film 13 mm × 17 mm, which is acceptable even for mass production. Although a very small sensing area is required, fabricated IDC sensors provide high sensitivity to bacterial monitoring. Simple SRF and capacitance measurement, even in just two days, are indicators of the bacterial growth. Moreover, fabricated IDC sensors expressed specific patterns in the change in monitored electrical parameters (SRF, capacitance, charge transfer and solution resistance), enabling differentiation between *P. aeruginosa* and *S. aureus*.

## 4. Conclusions

In this paper, the nanocomposite based on SnO_2_-In_2_O_3_ was prepared using synthesized SnO_2_ and In_2_O_3_ powders, and 85:15, 90:10 and 95:5 weight percentage combinations of SnO_2_-In_2_O_3_ were processed using planetary ball-miller. The IDC bio-sensor based on SnO_2_-In_2_O_3_ nano composites were fabricated using screen printing technology. The sensing performances of SnO_2_-In_2_O_3_ thick-film sensors towards *P. aeruginosa* and *S. aureus* were studied using the impedance spectroscopic technique. The results obtained revealed the sensor’s dependency towards change in ionic concentrations in sensing bulk media and adsorbed potential ions on the surface of the electrode. Shifting of SRF to lower frequencies was observed ion Day II of the analysis compared to the data from Day I. This can confirm changes in the concentration of charged molecules and ions in sensing pathogen media. Moreover, SRF obtained from each pathogen system revealed individual SRF for each pathogen system. We observed that Rct and Rs values were lower on Day II of the analysis for both pathogen systems, using the proposed sensors.

The main contributions of this article can be summarized as follows: (1) synthesis and processing details of SnO_2_ and In_2_O_3_ mixtures are provided, (2) conducted structural characterization of fabricated nanocomposites confirmed regularity of our fabrication procedure as expected diffraction peaks, Raman shifts and electronic patterns are obtained, (3) sensing properties were analyzed on two consecutive days, confirming the expected impedance decrease during bacteria growth, and (4) sensing mechanism of fabricated sensors are analyzed and explained in detail, providing similarities and differences in the sensing of *P. aeruginosa* and *S. aureus* with the same SnO_2_-In_2_O_3_ mixtures.

Developed IDC sensors are based on economical and reliable fabrication procedures which have the potential for mass production. Moreover, readout electronics is based on impedance measurement which opens up a pathway to use affordable embedded hardware for measurement and data acquisition. Based on these features, a promising application of developed sensors can be bacteria identification in real biological samples, especially in real-world in situ applications where complex laboratory equipment is not available.

Future work will be directed towards the development of nanomaterial-based sensors for application as flexible wearable sensing platforms of various pathogens.

## Figures and Tables

**Table 1:** Charge transfer (Rct) and solution resistance (Rs) values of SnIn5 based sensor.

Pathogen System	Rct (Ω)		Rs (Ω)	
	Day I	Day II	Day I	Day II
*P. aeruginosa*	0.21	0.10	20.45	18.28
*S. aureus*	0.091	0.084	12.83	11.68
